# Blockchain technology-based FinTech banking sector involvement using adaptive neuro-fuzzy-based K-nearest neighbors algorithm

**DOI:** 10.1186/s40854-023-00469-3

**Published:** 2023-03-10

**Authors:** Husam Rjoub, Tomiwa Sunday Adebayo, Dervis Kirikkaleli

**Affiliations:** 1 Department of Accounting and Finance, Palestine Polytechnic University-PPU, Hebron, Palestine; 2 Department of Banking and Finance, Faculty of Economics, Administrative and Social Sciences, Bahçeşehir Cyprus University, 99010 Alayköy, Nicosia, Turkey; 3 Department of Economics, Faculty of Economics and Administrative Science, Cyprus International University, Northern Cyprus, 10 Mersin, Nicosia, Turkey; 4 Department of Banking and Finance, Faculty of Economics and Administrative Sciences, European University of Lefke, Lefke, Northern Cyprus, 10 Mersin, Turkey

**Keywords:** FinTech, Economic growth, Blockchain technology, Adaptive neural fuzzy based KNN algorithm, Rolling window autoregressive lag modelling

## Abstract

The study aims to investigate the financial technology (FinTech) factors influencing Chinese banking performance. Financial expectations and global realities may be changed by FinTech’s multidimensional scope, which is lacking in the traditional financial sector. The use of technology to automate financial services is becoming more important for economic organizations and industries because the digital age has seen a period of transition in terms of consumers and personalization. The future of FinTech will be shaped by technologies like the Internet of Things, blockchain, and artificial intelligence. The involvement of these platforms in financial services is a major concern for global business growth. FinTech is becoming more popular with customers because of such benefits. FinTech has driven a fundamental change within the financial services industry, placing the client at the center of everything. Protection has become a primary focus since data are a component of FinTech transactions. The task of consolidating research reports for consensus is very manual, as there is no standardized format. Although existing research has proposed certain methods, they have certain drawbacks in FinTech payment systems (including cryptocurrencies), credit markets (including peer-to-peer lending), and insurance systems. This paper implements blockchain-based financial technology for the banking sector to overcome these transition issues. In this study, we have proposed an adaptive neuro-fuzzy-based K-nearest neighbors’ algorithm. The chaotic improved foraging optimization algorithm is used to optimize the proposed method. The rolling window autoregressive lag modeling approach analyzes FinTech growth. The proposed algorithm is compared with existing approaches to demonstrate its efficiency. The findings showed that it achieved 91% accuracy, 90% privacy, 96% robustness, and 25% cyber-risk performance. Compared with traditional approaches, the recommended strategy will be more convenient, safe, and effective in the transition period.

## Introduction

Today’s modern service expectations necessitate 24-h availability and international accessibility for all kinds of work. FinTech refers to the use of technology in global business structures to deliver better financial services to clients. However, the phrase itself is still a subject of debate. In actuality, FinTech is a blanket term encompassing several different technologies that constantly communicate in a common infrastructure (Xu et al. 2019). Traditional banks are under increasing pressure to update their core business operations and services as technology-driven enterprises that provide financial services become more prevalent (Bazarbash 2019; Fang et al. 2022). Many banks are now addressing digitalization concerns by partnering with startups that provide innovative banking services and unique service bundles (FinTech). Banking, traditionally one of the most conservative and traditional areas of the economy, has recently been challenged by potentially disruptive technology-driven innovations and Web solutions (Athari et al. 2022; Ayhan et al. 2022). By creating new information technology (IT)-enabled service models, new startup industries and worldwide technology companies in many contexts have created more customer-oriented and user-friendly automated processes in the banking industry, contributing to increased digital service innovation of financial products. FinTech companies have also played a role in the creation of many of these new banking products. Several of the latest technological advances can alter or potentially destroy the business operations of some of the more traditional institutions. As a result of digitalization and platform-enabled FinTech, banks have been compelled to review their organizational building and increase their accessibility to market interactions (Sebastião and Godinho 2021; Stojanović et al. 2021).

**Fig. 1:**
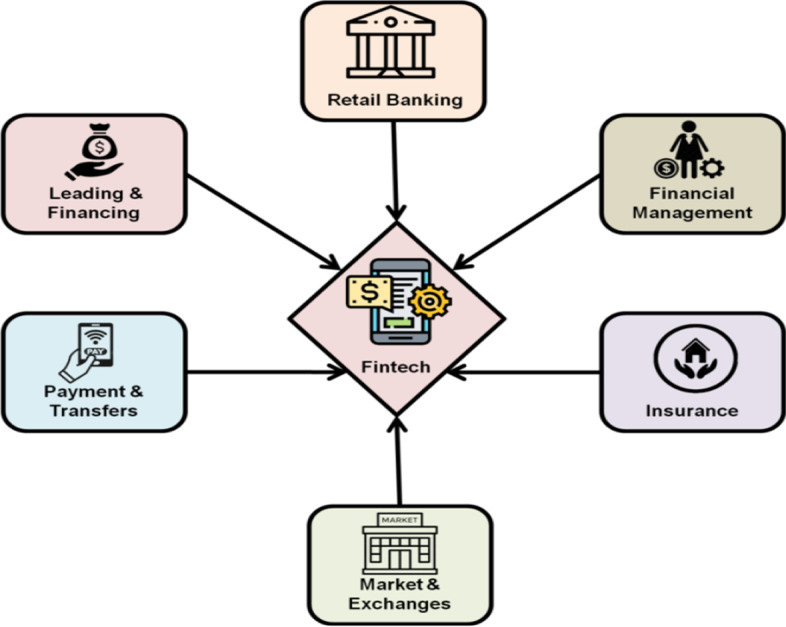
General representation of financial technology (FinTech)

FinTech offers identical services to banks, perhaps more effectively as a result of technology improvements, but in a distinct, and unbundled way. For example, crowdfunding platforms transform funds into lending in the same manner as banks. Unlike banks, however, they employ big data instead of long-term connections; access to care is only dispersed via online networks, risk and trust conversion are not conducted, and borrowers, and lenders, along with investors and investment possibilities, are immediately matched. These are all FinTech-related endeavors. However, the scope of these unmetered FinTech activities is limited. For example, platforms struggle to provide a diverse range of investment options to their customers without taking on some risk (Stojanović et al. 2021). Figure 1 shows a general representation of financial technology (FinTech).

FinTech payment systems have had a huge impact on the financial services industry. Payment systems or credit contracts are transitions that, unlike cars, or other purchases, generally do not involve any physical components. Another reason is that, apart from some physical involvement, such as customer assistance, many activities, such as payment processing or trading industries, are largely carried out without any actual touch (Claessens et al. 2018). The ongoing economic growth and development process is causing a substantial reorganization of the financial advisory production chain, including technological advances such as robo-advisors and new competitors entering the industry because of recent technological advancements, such as those of Apple. “Financial technology,” or “FinTech,” reflects growth induced by IT transformation (Li et al. 2022; Navaretti et al. 2018).

Cryptocurrencies are decentralized digital assets that enable safe electronic payments. The notion of cryptocurrency emerged as a division of the digital cash phenomenon, but it was only with the introduction of Bitcoin that it gained widespread acceptance. FinTech has witnessed several significant advancements over the last decade, with Bitcoin at the forefront. Furthermore, the worldwide business, and economic development crisis—which corresponds to the financial collapse that started in the US financial sector in 2007–2008—has reduced public trust in banking organizations, which has enabled cryptocurrencies to become available. Since then, the sector has grown rapidly, propelled by rising acceptability, increased media attention, steady inflows, the initial coin offering mania, and improved transition capacity. Cryptocurrency has the potential to open up new FinTech marketplaces. Cryptocurrency improves the efficiency of money transfers. FinTech benefits from cryptocurrency since it helps prevent fraud (Degerli 2019; Kou et al. 2021).

FinTech credit is a credit activity that is made possible by electronic platforms. Borrowers are normally connected directly with investors; however, other platforms lend straight from their financial sheets. FinTech engagement in the credit market and online credit use promote sales and transaction growth for global-level businesses. These factors show the extent of credit market frictions and the advantages of new credit technology in strengthening credit markets (Hau et al. 2019). A peer-to-peer (P2P) market is a networked model in which two individuals engage directly with one another to buy products and services or to jointly develop products and services without the need for a middleman or a commercial business. P2P lending platforms lure customers who have limited or no access to conventional banks' credit capabilities. Although many banks and financial institutions offer online loan application services, only a small number of them verify the applications (Najaf et al. 2022).

FinTech involves the new use of knowledge to solve the extensive catalog of transition issues that the insurance business is now experiencing. It incorporates the use of technology to enhance CRM, pricing consolidation, Omni channel marketing acquisition, the Digital Claims Process, and online policy purchase in customer interactions. In the case of blockchain, the use of car telemetry, sensor data, traceability, asset trading, and home security may help insurers generate new income streams. Wearable tech, genetic information, chronic disease management, and preventive healthcare may all change the way insurance companies serve their customers (Yang et al. 2021).

Several existing approaches to FinTech transaction, credit, and insurance systems have flaws. Some experts feel that the FinTech revolution is a double-edged sword: on the one hand, it reduces banking costs, while on the other, it adds costs for infrastructure that must be constructed to accommodate FinTech growth. According to Hinson et al. (2019), nonperforming loan risk has increased dramatically with growth in FinTech lending. The explosion in FinTech lending is a direct result of the FinTech revolution. It has increased the likelihood that people and families can obtain loans at reasonable rates. According to Claessens et al. (2018), the low collateral requirements, and insufficient legal backing in FinTech are to blame for the rise in nonperforming loans. However, Lee and Shin (2018) stated that due to the short-term nature of these loans and their focus on the retail rather than the business sector, FinTech loan default risk is lower than that of a traditional commercial bank loan. Some studies have shown that the banking industry has become more vulnerable to competitive pressures because of the rising impact of FinTech. For instance, FinTech expansion has been shown to decrease bank market share, increase banking regulations, and lower bank profit margins, among other negative effects.

This paper introduces a blockchain-based finance solution for tackling various transition challenges within the banking sector. First, payment, credit, and insurance are gathered for the dataset. The dataset is then denoised for outliers/noises through the normalization approach. These normalized data are stored in the blockchain through smart contracts to prevent cheats. Due to the limitations of existing approaches, the rolling window autoregressive lag (RARDL) modeling approach is suggested in this research to examine economic improvement. The study contributes to the ongoing literature because blockchain technology is anticipated to improve data protection, help accelerate resolution, and automate processes to reduce expenditures. Open banking may leverage payment systems enabled by blockchain. However, if properly used and controlled, the self-governing capabilities of blockchain might result in systemic changes. Higher hardware prices and computer power requirements could hinder its deployment.

The remaining parts of this paper are the (1) literature review and problem statement, (2) proposed work, (3) performance analysis, and (4) conclusion.

## Synopsis of past studies

Le et al. (2021) review the linkages between the expansion of FinTech loans and the efficacy of financial institutions. According to the study, they appear to have a two-way connection. Consistent with a negative relationship between bank profitability and FinTech credit, FinTech credit is more developed in countries with less efficient banking systems. Meanwhile, the positive impact of FinTech credit on banking system efficiency suggests that FinTech credit might be a wake-up call for the banking system. Thus, governments worldwide should promote FinTech credit. Moreover, Najaf et al. (2022) examine recent advances within the FinTech industry and the resulting transition issues. They draw on current BIS work, including recent FSI research on national and global policy measures to adapt existing financial regulations to new activities and participants. Payment, investment management, fundraising, borrowing, equity markets, and insurance services are the six FinTech marketing strategies employed by a growing number of FinTech companies. FinTech refers to a broad set of technological advances facilitated through technological advances in the delivery of services to the authorized customer, economic organization technicians' internal manufacturing operations, and the layout of market enterprises without installing extra potential configurations of intersectional activities at risk (Hinson et al. 2019).

In addition, Hernández et al. (2019) demonstrated that FinTech has shown its importance as an innovation-driven field in the modern financial industry and is capable of transforming traditional financial marketing, providing a basic overview of FinTech and its growth in Vietnam. The research also includes a survey of expert opinions on the roadblocks to establishing FinTech programs to modernize Vietnam's banking and finance sector. Moreover, Bhasin and Rajesh (2021) explain the dramatically changing Indian banking system, which is moving away from traditional banking toward the e-collaboration of digital bank services and FinTech enterprises. The new structures are generating financial disruptions and changing the payment system structure. On the other hand, FinTech firms, while technologically advanced, must create client confidence for new digital and FinTech products to be adopted. The many problems and possibilities that the Indian banking industry faces in cooperation and coinvention with FinTech startups are detailed in this study report.

Thakor (2019) examines FinTech and its relationship with banking. The study begins with a description of FinTech, then examines various figures and stylized facts, and finally reviews conjectural, and experimental journalism. Hinson et al. (2019) analyze poor nations, finding that to fully profit from FinTech's promise within this environment, significant restrictions, and hazards must be overcome. Massive infrastructure expenditures and large-scale capacity growth are two mitigating variables. Appropriate policy recommendations require a thorough study of the financial stability and cost-effectiveness of emerging models for FinTech. The study reported that increases in green financing activity positively affect environmental and economic growth.

Williams (2021) examines the threat FinTech poses to traditional financial institutions and the advantages and hazards of financial democratization. Because analyzing the impact that FinTech has on market structures is difficult because of a lack of established data, the second section of this chapter combines relevant data from various sources with theoretical model predictions and evidence-based and questionnaire evidence to shine a light on how marketplaces are developing and group members are behaving. A study by Degerli (2019) reported that companies, regardless of industry, reconfigure their services in today's environment, with competitive circumstances continually changing.

Moreover, Pinshi (2021) examined the probable impact of FinTech on the financial system during the dark period of COVID-19. The study outcome shows that in that time of financial turmoil, FinTech seemed to see itself optimistically as a rebalancing mechanism for the global business financial system and the greatest tool for enhancing economic growth and development program adaptability and reacting to the emergency by ensuring system functionality while complying with countermeasures and trying to prevent the virus from spreading. Furthermore, Sheikh et al. (2019) discussed the financial sector's progress, prospects, and problems in China resulting from new technology. This chapter examines the possibilities arising from a large population, high penetration, and access to the most up-to-date and cheap technology, as well as government rules and regulations. The study findings show unrealized FinTech potential in China. The reality is that the financial system develops new features, attributes, and components as a result of globalization, technological development, and innovations as new financial instruments and inventions enter the financial environment and demand evolves.

Navaretti et al. (2018) provide a study agenda to predict future services research into the digitization of financial advice systems through FinTech—novel products developed by new rivals that threaten the existence of established financial institutions. This study introduces a use-inspired research program that integrates management challenges connected to FinTech with services marketing literature to provide intellectual and institutionally relevant research choices. The microlevel intricacy of digitized financial advisory networks, the microcoordination of value cocreation with FinTech, and the evolution of elastic structures, models, and markets are all on the table.

The Financial Services Authority (OJK) is responsible for safeguarding the public from fraudulent online FinTech loan networks (P2P lending) through public administration. Online loans have become more common as the COVID-19 epidemic has progressed, and OJK plays a critical role in preventing and safeguarding the public from illicit online loans through public administration. OJK promotes education and socialization so consumers can make better-informed decisions about online loans (Aziz and Nur’aisyah 2021). Similarly, Sheikh et al. (2019) stated that FinTech innovation is a hot issue globally. FinTech startups include lenders, cryptocurrencies, payments, and personal financial management, to name a few. Here, we review certain academic research before classifying well-known Iranian FinTech firms.

Due to the potential consequences of continued use of this new tech, blockchain technology is presently one of the most relevant subjects for academics and business (Fernandez-Vazquez et al. 2022). FinTech firm adoption of this method represents the next stage in ensuring blockchain longevity and growth. A paper by Tsai et al. (2016) presented the super-large ledger (SLL), a novel trade-clearing structure that may be deployed among exchangers, bankers, and authorities. As demand grows, the SLL will be divided and distributed across other computers to make the process more efficient. Carta et al. (2020) offered a generic approach to the well-established technical trading investment strategy using various logistic regression and dynamic asset choices. The overall resource evaluation uses a quality control method to determine whether a function carrying equities has shown subpar prediction performance before using it as an entry in the exchange process.

Likewise, Chen and Liao (2021) investigate the state of progress, future possibilities, and planning processes for finance and Internet technologies. FinTech employs several technical advancements, including big data, and cloud computing, to enable technology to support the finance sector and significantly improve production efficiency. To achieve knowledge transfer, the Internet of Things links everything to machinery. Hernández et al. (2019) analysed recent developments in this field and data security implementation in financial planning systems. Due to the high degree of protection that FinTech demands, the study also evaluates information security growth within that industry. Additionally, it reviews data storage difficulties in the cloud and encryption technologies for guaranteeing information security.

The banking industry has sizable revenue, earnings, and volume, posing both possibilities, and problems. In the digital age, it has experienced a period of transition in consumer and personalization terms. Additionally, the key participants are the consumers. For sustained development, their contentment is crucial. FinTech development has encountered several difficulties despite early enthusiasm about it. Due to the risk of hacking and concerns about the disclosure of private consumer financial information, data security is a major barrier for FinTech organizations. The hefty startup costs for financial businesses are another area of difficulty. We thus designed an adaptive neuro-fuzzy-based K-nearest neighbors algorithm (ANF-K-nearest neighbors (KNN) to identify the mismatch and successfully integrate it with banking to evaluate the impact on customer satisfaction as a means of resolving this problem.

## Proposed work

**Fig. 2:**
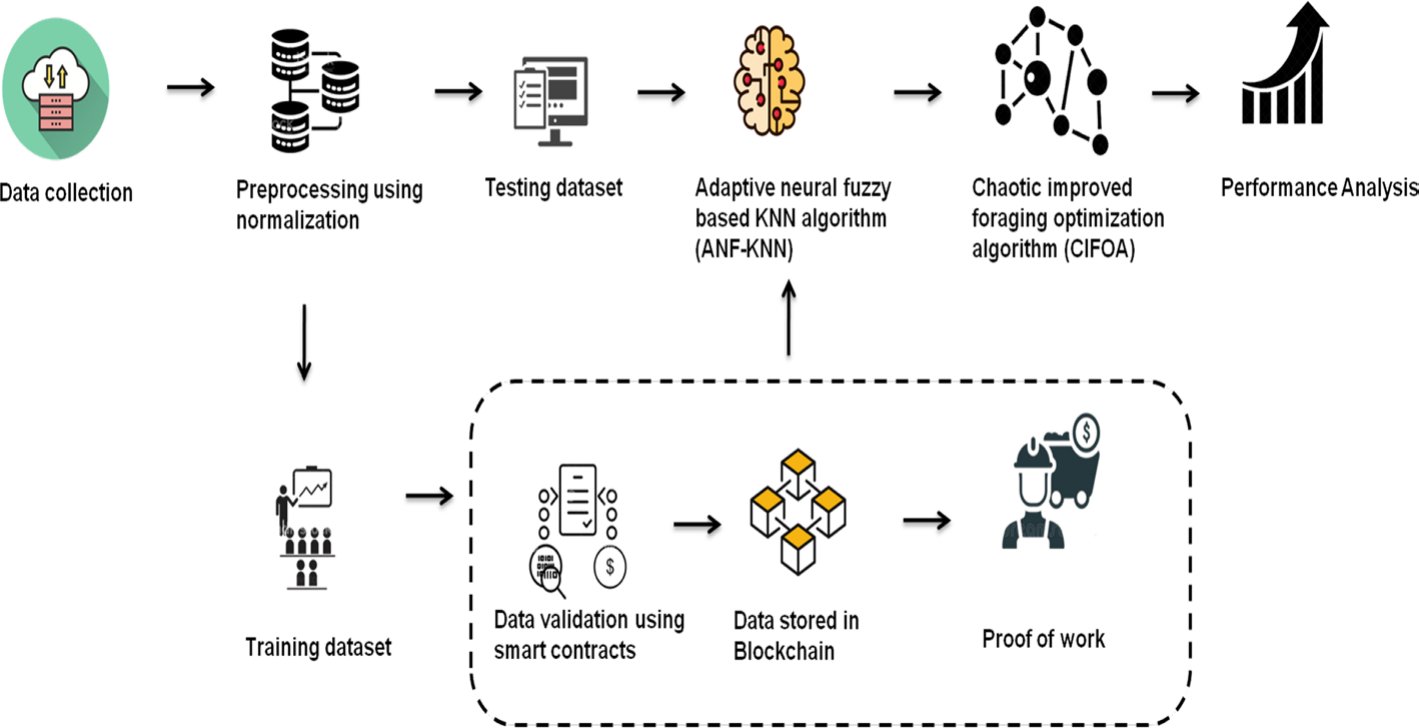
Overall representation of the proposed work

This paper provides a new method to overcome transition issues with the existing methods used for FinTech payment systems, credit markets, and insurance systems. The suggested technique often shows better performance with continuous data than that of the genetic and particle swarm algorithms. The suggested technique references previously collected data to classify a network. The proposed method will be more comfortable, secure, and effective than the genetic and particle swarm algorithms. Blockchain-based FinTech is proposed for predicting attacks within the banking sector. The ANF-K-nearest neighbors (KNN) is presented in this study. We have also proposed a chaotic improved foraging optimization algorithm (CIFOA). Figure 2 provides an overall representation of the proposed work.

### Data collection

Using a dataset of 28 Chinese banks from 2009 to 2018, both “linear and nonlinear” associations between corporate social performance and banking performance were found. Payment-focused FinTech firms can recruit clients rapidly and at a reduced cost, and they are among the most innovative and quick in adapting to new payment services. Consumer and retail payments and wholesale payments are the two marketplaces for FinTech payment data. A broad range of credit market services, including investing, foreign currency, trading, regulatory compliance, and research, influence FinTech firm models. Capital markets trading is a potential FinTech area. Trading FinTech allows traders and investors to connect in real time to consider and share information, buy and sell items and securities, and manage risks. A further capital markets idea for FinTech marketing is foreign currency transition. In FinTech insurance business strategies, FinTech aims to allow for more direct engagement between the insurer and the customer. As the pool of potential clients develops, consumers are offered solutions that meet their needs. They evaluate and correlate risks using data analytics.

### Data preprocessing using normalization

Data preprocessing entails the preparation and transformation of collected data into an appropriate format. Preprocessing data seeks to minimize data size, establish data relationships, normalize data, eliminate outliers, and extract data characteristics. Data cleansing, integration, transformation, and minimization are some of the strategies used. The primary goal of data normalization is to reduce or eliminate redundant data. Min-mix normalization is a method for performing linear transformations on a set of data. This is a technique for keeping the original data connected. Min–max normalization is a simple method for fitting data inside a predefined boundary:

1 $$U^{,} = \left( {\frac{U - min\;value\; of\; U}{{max\;value\; of\; U - min \;value \;of\; U}}} \right)*\left( {V + D} \right) + D$$

Here $U^{,}$ are the min–max normalized data, [V, D] is a predefined boundary, and U is the original data range.

F-score normalization is a method that uses concepts like quantitative variables to obtain normalized values or sets of information from unstructured data. As a result, the F-score parameter may be used to normalize unstructured data, as presented in the following equation:

2 $$g_{i}^{^{\prime}} = \frac{{g_{i} - \overline{J}}}{std\left( J \right)}$$

$g_{i}^{^{\prime}}$—F-score normalized one’s value, $g_{i}$—the value of row $\overline{J}$.

3 $$std\left( J \right) = \sqrt {\frac{1}{{\left( {k - 1} \right)}}\mathop \sum \limits_{l = 1}^{k} \left( {g_{i} - \overline{j}} \right)^{2} }$$

4 $$\overline{j} = \frac{1}{k}\mathop \sum \limits_{l = 1}^{k} g_{i}$$

The technique that gives a scale from − 1 to 1 is known as decimal scaling. From the decimal scaling procedure, as a consequence,

5 $$g^{i} = \frac{g}{{10^{n} }}$$

$g^{i}$-scaled value, $g$-range of values, n-smallest integer Max ($\left| {g^{i} } \right|$) < 1.

Using min–max normalization, the original data are converted linearly. Assume that min_n_ and max_n_ are the lowest and maximum values for variable *P*. By computing [new-min_n_, new-max_n_, a value e of P is transferred to p' within the range through min–max normalization [new-min_n_, new-max_n_].

6 $$P\prime = \left( {\left( {p - min_{n} } \right)/\left( {max_{n} - min_{n} } \right)} \right)*\left( {new - max_{n} - new - min_{n} } \right) + new - min_{n}$$

The values for variable P are normalized using the mean and standard deviation of P in w-score normalization. P-value e of P is normalized to pʹ using the following formula:

7 $$p\prime = \left( {\left( {p - \overline{N}} \right)/\sigma_{N} } \right)$$

where p and $\sigma_{N}$ are the mean and standard deviation of variable e, respectively. When the real minimum and maximum of variable e are unknown, this approach of normalizing is beneficial.

The decimal point of values of variable p is moved during normalization using decimal scaling. The number of decimal points shifted is determined by an absolute maximum value. A value p of *P* is normalized to pʹ using the following formula:

8 $$p\prime = \left( {p/10^{a} } \right)$$

where i is the smallest integer such that Max($\left| {p\prime } \right|$) < 1.

The preprocessed data is called the training data set. These data are validated using smart contractors. Then it will store in the blockchain. Testing data set is directly tested through the proposed algorithm.

### Adaptive neuro-fuzzy-based K-nearest neighbors algorithm

The ANF-KNN is a data categorization method that calculates the probability that a data point belongs to one of two groups based on determining to which group the data points closest to it belong. The KNN method does not create a framework using the training set until the data set is queried.

The ANF-KNN gives a more accurate membership vector for the objects, as well as taking into consideration the intensity of object membership. When using this strategy, a group of objects is assigned to the most common group based on its KNNs. The ANF-KNN assigns sample fuzzy memberships and aids decision-makers in making fuzzy judgments.

The ANF-KNN divides (clusters) the example vectors Y = {y1, y2,..., y_m_} S_m_ into k(1 < k < m) fuzzy subsets. The fuzzy membership matrix is illustrated as A for p = 1, 2,..., kand q = 1, 2,..., m, where U_pq_ represents the fuzzy membership degree of yq in class p. In a nonfuzzy variant of the procedure, the *q*th item is assigned to the *i*th class with the biggest A_pq_ with respect to the degree of adaptive neuro-fuzzy membership of all the other classes. The restrictions on matrix A are as follows:

9 $$\mathop \sum \limits_{c = 1}^{k} a_{cd} = 1, \;d,$$

The initial requirement in Eq. (9) guarantees that all entity degrees of membership are attained across all groups p = 1,2,…,k, and that the sum of all degrees of membership is one.

10 $$a_{cd} \hat{C}\left[ {0,1} \right], 0 < \mathop \sum \limits_{d = 0}^{k} a_{cd} < m$$

A few of the entities' fuzzy degrees of membership are in the region of comparability of more than zero to less than one, as shown in Eq. (10). The two conditions show that if an object belongs to a group with A = 1, it has a membership degree of zero in all other classes.

In the ANF-KNN, each vector is given a fuzzy degree of membership depending on the ranges of their KNN membership, as follows:

11 $$A_{c} \left( Y \right) = \frac{{\mathop \sum \nolimits_{d = 1}^{f} \left( {\frac{{a_{cd} }}{{Y - Y_{d}^{{\frac{2}{b - 1}}} }}} \right)}}{{\mathop \sum \nolimits_{d = 1}^{f} \left( {\frac{1}{{Y - Y_{d}^{{\frac{2}{b - 1}}} }}} \right)}}$$

where K is a fixed variable and m is a specified number of closest neighbors. The option m defines the degree of each nearest neighbor in the calculation of the fuzzification value.

However, the Nash–Sutcliffe model efficiency coefficient (*E*_*NS*_) may be used to determine how reliable prediction models are in practice. The Nash–Sutcliffe coefficient is defined as follows:

12 $$E_{NS} = 1 - \frac{{\mathop \sum \nolimits_{c = 1}^{m} \left( {g_{c}^{*} - g_{c} } \right)^{2} }}{{\mathop \sum \nolimits_{c = 1}^{m} \left( {g_{c}^{*} - G} \right)^{2} }}$$

This coefficient ranges from − ∞ to 1, with a value near 1 denoting greater accuracy, a value of exactly 0 denoting that the forecasting algorithm is as accurate as the observed data mean, and any negative sign identifying that the predictive model outcome is less accurate than even the observed data mean. This coefficient is a widely used statistic in research to determine the efficacy of a prediction model.

In the validation step of the models, five evaluation metrics are used to test the performance of the proposed ANF-KNN model: RMSRE, RMRE, CC, bias, and SI. The following are the definitions of these statistical measures:

13 $$RMSRE = \sqrt {\frac{1}{m}\mathop \sum \limits_{c = 1}^{m} \left( {\frac{{g_{c} - g_{c}^{*} }}{{g_{c}^{*} }}} \right)^{2} }$$

14 $$RMRE = \sqrt {\frac{1}{m}\mathop \sum \limits_{c = 1}^{m} \left| {\frac{{g_{c} - g_{c}^{*} }}{{g_{c}^{*} }}} \right|}$$

15 $$CC = \frac{{\mathop \sum \nolimits_{c = 1}^{m} g_{c} g_{c}^{*} }}{{\left( {\mathop \sum \nolimits_{c = 1}^{m} g_{c}^{2} \mathop \sum \nolimits_{c = 1}^{m} g_{c}^{*2} } \right)^{1/2} }}$$

16 $$Bias = \frac{1}{m}\mathop \sum \limits_{c = 1}^{m} g_{c} - g_{c}^{*}$$

17 $$SI = \sqrt {\frac{{\frac{1}{m}\mathop \sum \nolimits_{c = 1}^{m} \left( {\left( {g_{c}^{*} - G} \right) - \left( {g_{i} - G} \right)} \right)^{2} }}{G}}$$

where the observed and assessed pth are g_i_ and g_i_*, respectively, and m is the number of observations in the validating set of data. In the validation data set, G and G* represent the average observed and assessed, respectively.

**Table:** 

Adaptive neuro-fuzzy-based K-nearest neighbors algorithm
*Start*
*Sample x should be entered*
*K *$(1\le K\le n)$* is the first set*
*Assign the integer i* = *1 as the preliminary value*
*The distance between the points x and xi should be calculated*
*If *$i\le K?$*, yes, In the list of KNNs, include *${u}_{i}\left(x\right)$
*Find the *${x}_{i}$* nearer to x than either neighbor before it*
*Include *${x}_{i}$* in the KNN collection*
*Then eliminate the KNN who is farthest away*
*i* = *i* + *1*
*Is it possible to identify KNNs relative to x?*
*Set i* = *1 as the initial value*
*Use Eq. (**3**) to calculate *${u}_{i}\left(x\right)$
*Compute *${u}_{i}\left(x\right)$* using Eq. (**11**)*
*Is it amusing that all courses' membership degrees are determined?*
***End***

### Chaotic improved foraging optimization algorithm

The CIFOA is a global optimization technique for optimization and control. The CIFOA is a new optimization algorithm 

### Rolling window autoregressive lag modeling

Besides autoregressive distributed lag (ARDL), major rolling window mechanisms have been used in many kinds of research. The ARDL assumption implies that the lagged vector and parameters remain constant throughout the time series. However, if this is not the case, ARDL modeling could cause us to reach an absurd conclusion. The assumption of consistent cointegrating vectors and variable stability must be loosened to reflect the underlying nature of the connection. RARDL can help with this since it relaxes the assumption; even if the assumption is correct, it also provides strong proof. RARDL modeling is used to analyze economic growth.

Let the size of the rolling window be K_s_ for a sample population of K. The following steps are used to perform the RARDL bounds test:

*Step 1* For k = 1, 2,…, K_s_, the ARDL bounds test is performed on the first K_s_ samples of A_k_ and B_k_. D stat, q-value, and other statistics of importance are reported.

*Step 2* An ARDL bounds test is performed by removing the initial observations and adding observation, i.e., the (K_s_ + 1)th of K_s_ and B_k_. That is, for k = 2, 3,…, K_s_ + 1 samples of A_k_ and B_k_, the ARDL bounds test is approximated. D stat, q-value, and other relevant statistics are mentioned once again.

*Step 3* Step 2 is performed until all of the remaining observations T have been accounted for. As a result, (K-K_s_ + 1) ARDL limits serial correlation tests, and their comparative statistical data will exist.

## Performance analysis

This paper provides recommendations for improving FinTech within the banking sector, including payment systems (cryptocurrencies), credit markets (P2P lending), and insurance systems. This article discusses using FinTech within the banking sector based on an ANF-KNN employing blockchain technology. We review FinTech involvement within the banking sector in terms of investment range, transaction speed, credit market development, insurer satisfaction, and accuracy. These parameters of the proposed method are evaluated and compared with existing optimization methods such as the K-means clustering algorithm (K-CA), multiple criteria decision-making method (MCDM), greedy algorithm (GA), and decision tree algorithm (DTA).

### Growth in FinTech—bank alliances

**Fig. 3:**
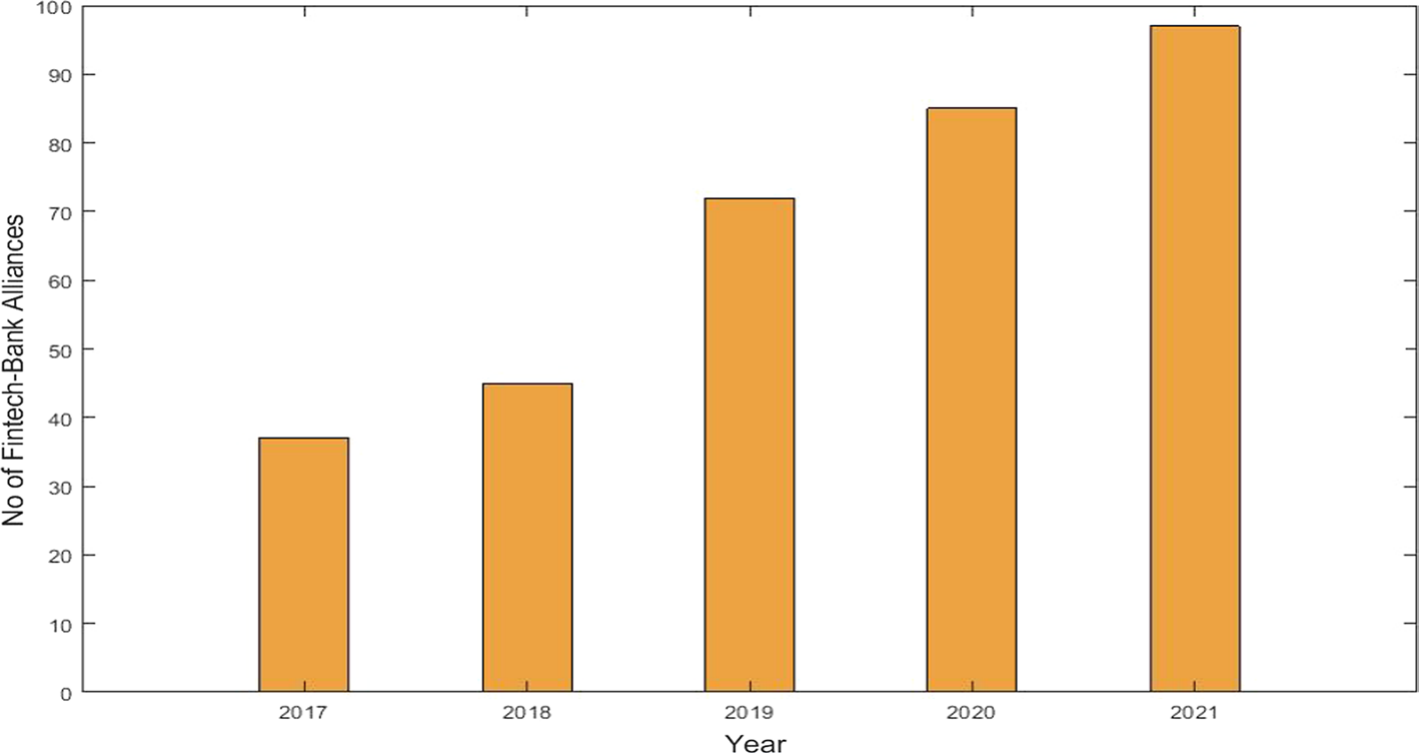
Growth of FinTech in banking

FinTech—bank alliances involvement in recent years is growing higher because of making transactions with convenience. Bank customers will have the opportunity to transact through cutting-edge technology, saving time, effort, and money. High transaction volumes with minimal operational costs in a short period will also benefit banks and FinTech. Figure 3 depicts the growth in FinTech—bank alliances from 2017 to 2021.

### FinTech growth in the banking sector

**Fig. 4:**
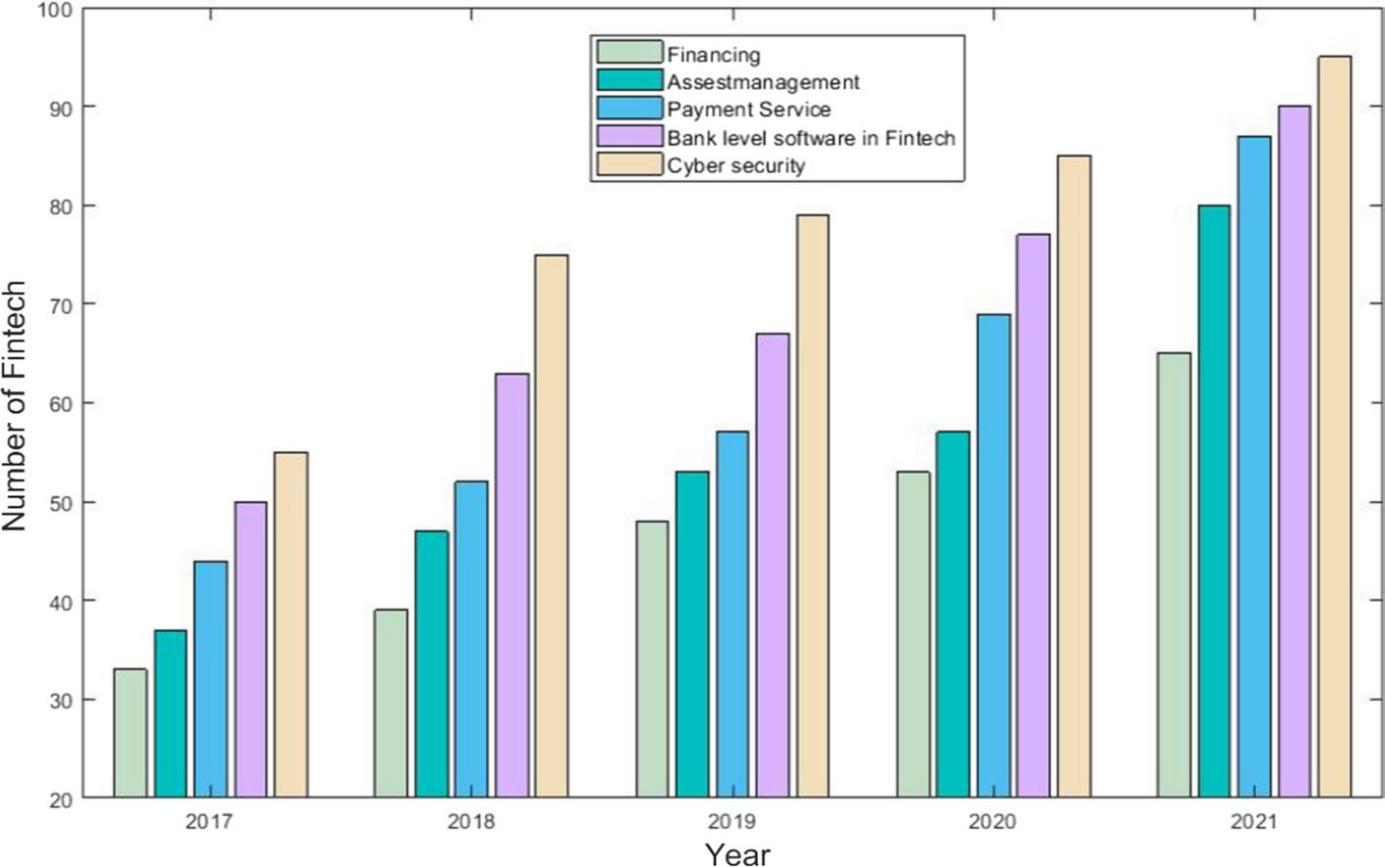
Financial technology (FinTech) banking sector activities

FinTech involvement in the banking sector adds to the financial services and security of transactional activities. The five key banking activities seeing growth due to FinTech involvement are financing, asset management, payment services, bank-level FinTech software, and cybersecurity. Figure 4 shows the growth in these FinTech-related activities, with FinTech involvement at banks rising rapidly from 2017 to 2021.

### Credit market development

**Fig. 5:**
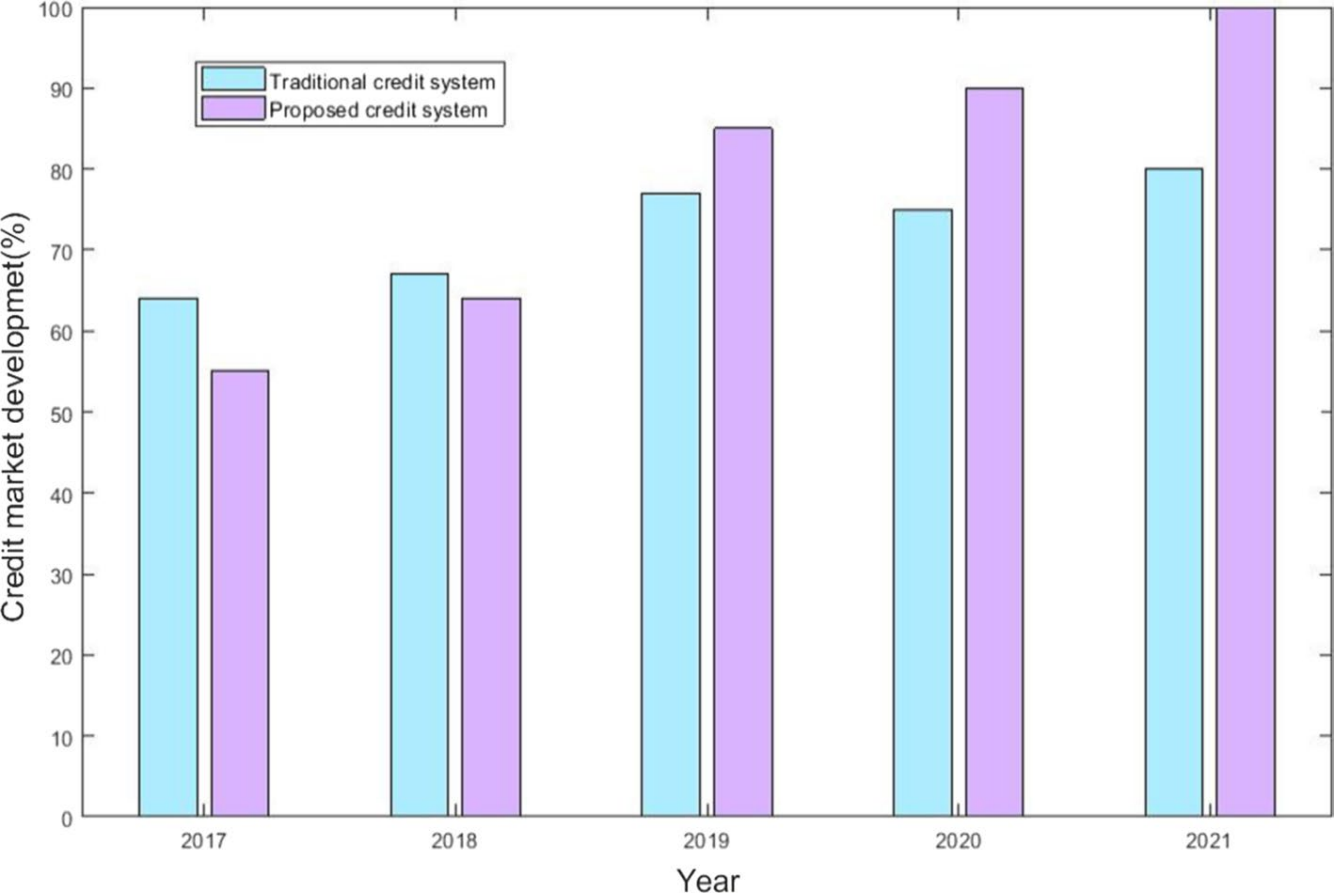
Credit market development

FinTech credit activity is made possible by the online platform. Borrowers are normally connected directly with investors; however, other platforms lend using their balance sheets. FinTech platforms provide a variety of credit types, including consumer, and commercial lending, real estate loaning, and trade finance. Due to the benefits for credit markets, FinTech use is increasing. Figure 5 shows the comparison of the credit system for the proposed method and the traditional method. The figure shows that compared with a traditional credit system, the proposed credit system developed with FinTech grew rapidly from 2017 to 2021.

### Economic growth

**Fig. 6:**
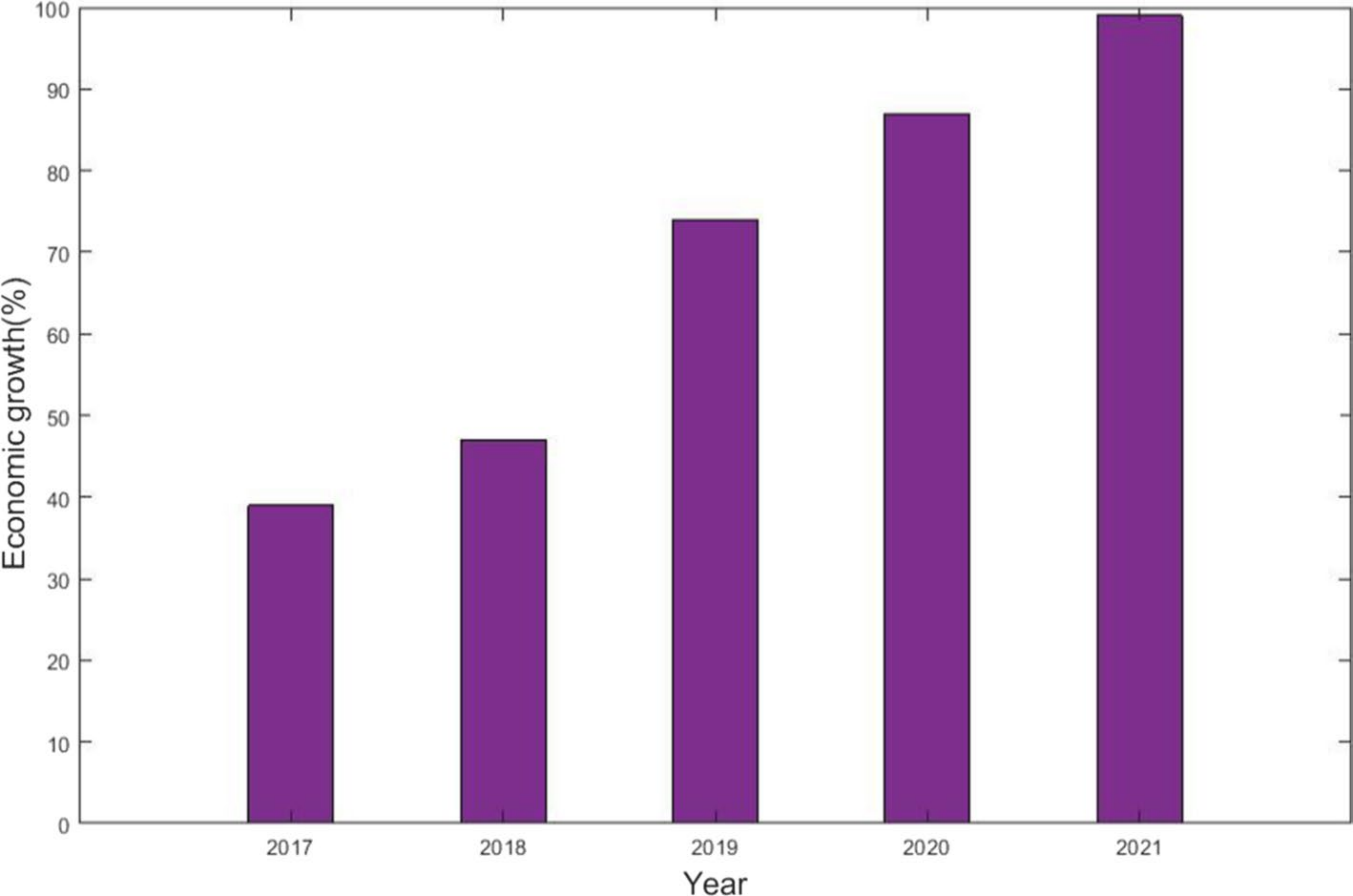
Economic growth (%)

By increasing the gross domestic product generated by the financial industry and indirectly boosting e-commerce sales and real estate financing, specifically by decreasing loan rates for all enterprises, FinTech advancement contributes to the economic development sector, as well as indirectly enhancing e-commerce turnover and real-sector finance, particularly by reducing lending rates for all companies. Figure 6 shows the economic growth from FinTech. The figure clearly shows that the economy was growing rapidly from 2017 to 2021 due to FinTech advancement.

### Investment range

**Fig. 7:**
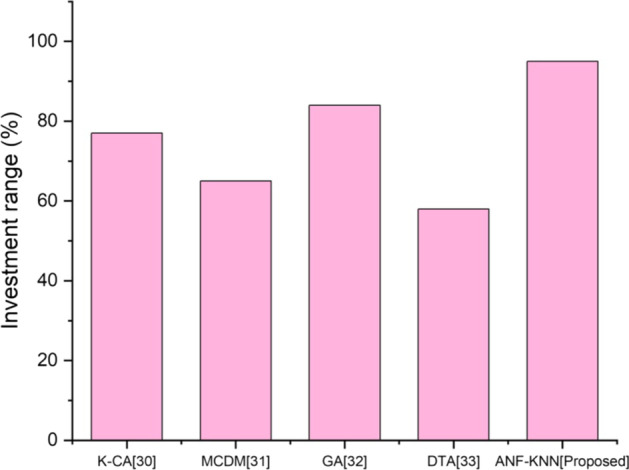
Investment range

The proposed method will change the landscape of investment management. Because of the technologies provided in blockchain-based FinTech, involvement is used to assess investment possibilities, portfolio optimization, and risk mitigation. Figure 7 shows the comparative analysis of the proposed method's investment range with existing methods. The figure shows that the amount invested in implementing the proposed method is higher than with traditional methods.

### Scalability

**Fig. 8:**
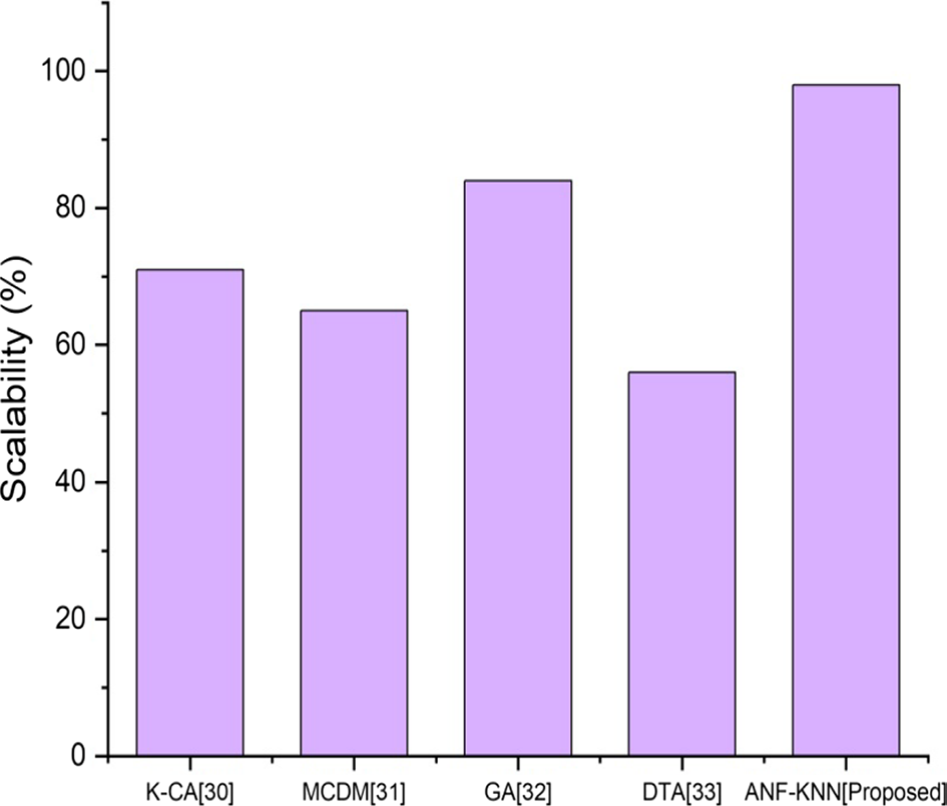
Scalability comparison of existing and proposed method

Scalability in financial markets refers to a financial institution's capacity to manage rising market demands. In the corporate world, a scalable corporation can maintain or enhance profit margins as sales volume grows. In a comparative analysis, the proposed method provides more scalability than other existing methods. Figure 8 shows a comparative analysis of the proposed and existing methods. The figure shows that the scalability of the proposed method is superior to that of conventional approaches.

### Accuracy

**Fig. 9:**
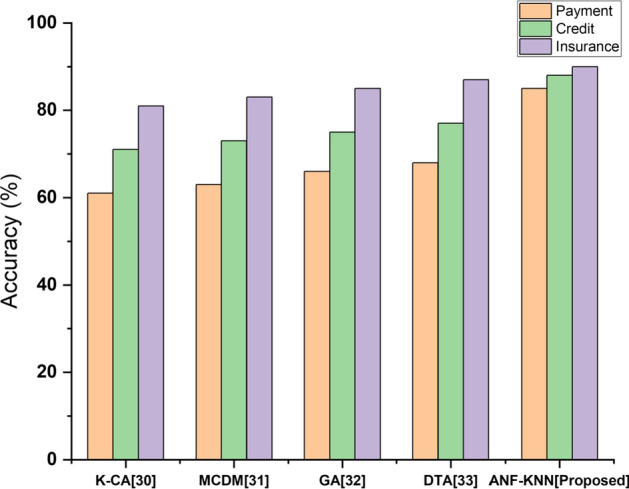
Accuracy comparison of existing and proposed method

The proposed method of FinTech involvement within the banking sector must be more accurate for payment systems, credit markets, and insurance systems. For these three platforms, the process will be safer, more efficient, and more convenient. Figure 9 shows a comparative analysis of the accuracy of the process involving FinTech. The figure shows that the accuracy of the proposed method is higher than that of the existing method.

### Privacy

**Fig. 10:**
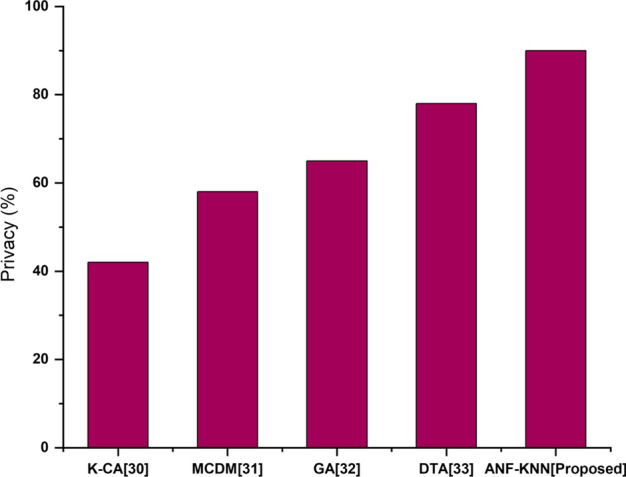
Privacy comparison of existing and proposed method

Customers require that their personal information and financial transaction history are kept private. Customer identifying information, such as credit information, payment history, bank account authentication information, and Social Security numbers, are kept private much more efficiently with the proposed technology than with others. FinTech companies reduce risk in the digital environment by using proactive cybersecurity services. Figure 10 shows a comparative analysis of privacy for a proposed method and existing method. The figure shows that the privacy of the proposed method is superior to the existing method.

### Robustness

**Fig. 11:**
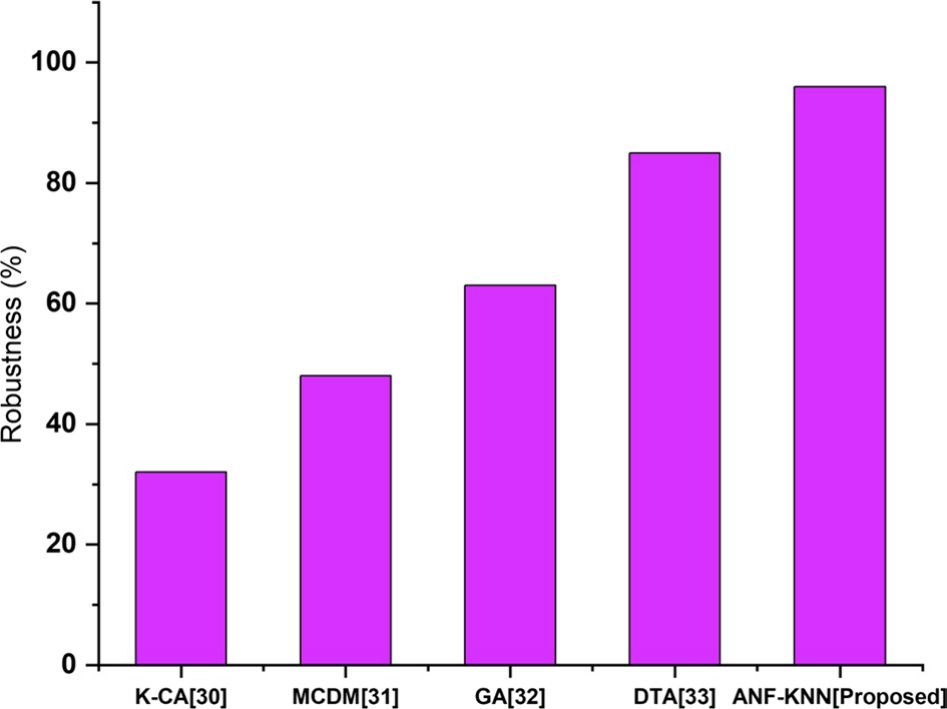
Robustness comparison of existing and proposed method

Robustness is a property that describes a model’s, test’s, or system’s capacity to work well when its parameters or hypotheses are changed in the investing world. A solid notion will work without fail and deliver great outcomes in many situations. The proposed FinTech system technology is highly robust compared with other existing FinTech systems. Figure 11 shows a comparative analysis of the robustness of the proposed and existing methods. The figure shows that the proposed method has higher robustness than the existing method.

#### Cyber-risks

**Fig. 12:**
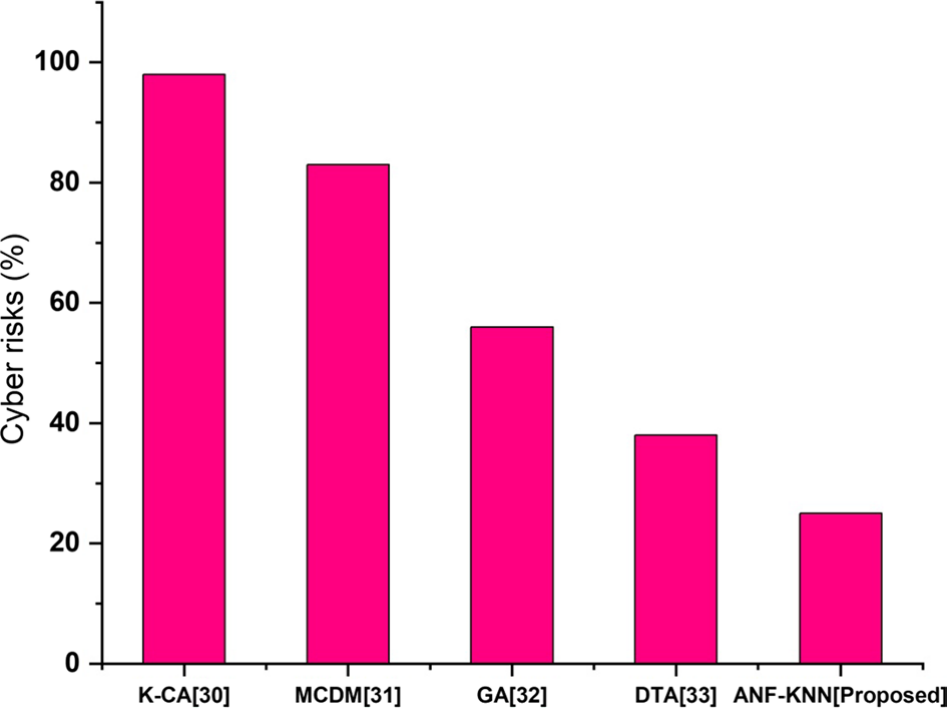
Cyber-risk comparison of existing and proposed method

Vulnerable to unauthorized access, intimidation, denial of service attacks, and credit card fraud have all become common financial cyber-risks. These cyberattacks have the potential to put the financial system in danger. The proposed method will deny these types of cyberattacks in FinTech. Figure 12 shows the comparative analysis of cyber-risks for a proposed method with existing methods. The figure shows that the proposed method has more risk than the existing method.

#### Discussion

Blockchain is a decentralized network ecosystem with a digital ledger in which transactions are easy for the customer. It offers protection, safety, accessibility, and identity via a set of instructions and cryptographic methods. The proposed blockchain-based ANF-KNN and CIFOA focus on the challenges of FinTech involvement within the banking sector, such as investment range, scalability, accuracy, privacy, robustness, and cyber-risks. This paper has compared these challenges with four other existing technologies, namely K-CA, MCDM, GA, and DTA.

Investment in the FinTech-based banking sector is mainly based on trust and the advancement of technology in financial activities. The investment banking sector has struggled to adapt to legacy technology, but as the digital revolution sweeps across finance, the moment to modernize is now. FinTech will change investment banking in several ways, including using sophisticated technology. Investment institutions must adapt and embrace these technological advancements to stay competitive. Figure 7 shows the high-level investment range of FinTech using the proposed method. One of the most significant issues with existing methods is scalability. Today, increasing network size is seen as a changing objective rather than a goal. A key goal of cryptocurrencies is to achieve a per-second transaction rate comparable to that of a centralized system while holding the underlying technology constant. There is a need to establish a blockchain that can handle scalability and develop a network of computers that can confirm each transaction. Figure 8 shows the higher scalability of the proposed method compared with existing methods. Accuracy is defined as the efficiency and effectiveness of the process using FinTech. The existing system poses some issues for FinTech. The proposed method will be more effective for all financial transactions and other activities that involve FinTech.

Figure 9 compares the accuracy of the proposed and existing methods. Privacy is the most serious challenge for existing financial activities, and existing methods experience more issues in maintaining customer data privacy. The transparency of financial activities, one of the technology's core strengths, could become an enormous challenge. Due to economic and client privacy implications, two firms dealing with each may be hesitant to have this information monitored by a third party. All customer personal data can be protected from hackers safely through the proposed method. The privacy level of comparative analysis for the proposed and existing methods is shown in Fig. 10.

When the proposed technology exhibits features comparable to those of existing economic networks after overcoming the aforementioned problems, the system has achieved resilience. Nonetheless, today's technology is confronted with issues that must be solved. Robustness is measured by increased availability, which means no downtime, and transaction services that are continuously available. Based on comparison analysis, Fig. 11 shows the robustness of the proposed method. Banks and other financial institutions all around the globe have been the subject of cyber-risks.

Cybersecurity concerns influence practically every aspect of the financial industry. They might put financial institutions that employ technology, FinTech companies, and financial clients in the FinTech ecosystem at risk. Additionally, technology engineers must be aware of possible cybersecurity threats that might exploit vulnerabilities and weaknesses in the proposed technology. Malware, data breaches, interruption of service, cybercrime, and hacking are just some of the cyber-risks to which existing FinTech has been exposed. The two most prevalent cyberattacks documented regularly in the chronology of cyber-hazards and threats on FinTech worldwide are unauthorized access and distributed denial of service. The proposed method protects against these cyber-risks in financial activities, as shown in Fig. 12.

## Conclusions

In conclusion, conventional transition methods, qualifying for and receiving a loan from a finance business, and insurance terms, and policies are significant obstacles to contemporary technology and could affect future economic growth and development. This research provides a novel insight into the appropriate functioning of the AFN-KNN algorithm, which is based on blockchain technology. To enhance all transactional, lending, and insurance processes securely, and effectively, we have suggested the CIFOA. This enhancement would promote the expansion of economic organizations and industries. The suggested technique achieves 91% accuracy, 90% privacy, 96% robustness, and 25% cyber-risk. The digitalized FinTech-based process will provide people the chance to transact while making use of the newest technologies and saving time, effort, and money. As a result, the suggested solution will also benefit FinTech, since it will quickly result in a high transaction volume with low operating expenses.

Because it has changed how businesses function, FinTech is presently well liked. Consumers may now transfer money online much more easily due to this industry. FinTech companies were created in response to the need for a more effective financial system with real-time applications. Furthermore, due to the impact of the pandemic, many firms were better able to understand the significance of FinTech and are now able to advise their customers about how to satisfy their financial needs as technology advances. We believe that the number of FinTech-related patent applications will continue to increase due to the importance of applying FinTech to develop digital finance within the financial sector.

In future research, investigators may collect more FinTech datasets to examine the impact of FinTech in more detail. They could further subdivide the data into subsamples depending on financial region, size, ownership, and other factors. Future FinTech development could also focus on several diverse sectors, such as risk management, precise financial distribution, loan management, and client maintenance, and services. The findings suggest that to improve performance, the financial sector should exert greater effort when filing for FinTech patents. However, there are limitations to this research. First, the study was limited to the financial and banking industry within China. Second, because of the small sample size, several methodological issues with the study might limit the validity of the empirical findings.

## Data Availability

The data that support the findings of this study are available from World Bank.

## Abbreviations

IoT: Internet of things
AI: Artificial intelligence
ANF-KNN: Adaptive neural fuzzy based KNN algorithm
CIFOA: Chaotic improved foraging optimization algorithm
DTA: Decision tree algorithm
DdoS: Distributed denial of service
GA: Greedy algorithm
IT: Information technology
K-CA: K-means clustering algorithm
MCDM: Multiple criteria decision-making method
NPLs: Non-performing loans
P2P: Peer-to-peer
RARDL: Rolling window autoregressive lag modeling
SLL: Super-large ledger
